# Animal models for COVID-19 and tuberculosis

**DOI:** 10.3389/fimmu.2023.1223260

**Published:** 2023-08-11

**Authors:** Björn Corleis, Max Bastian, Donata Hoffmann, Martin Beer, Anca Dorhoi

**Affiliations:** ^1^ Institute of Immunology, Friedrich-Loeffler-Institut, Federal Research Institute for Animal Health, Greifswald-Insel Riems, Germany; ^2^ Friedrich-Loeffler-Institut, Federal Research Institute for Animal Health, Greifswald-Insel Riems, Germany; ^3^ Institute of Diagnostic Virology, Friedrich-Loeffler-Institut, Federal Research Institute for Animal Health, Greifswald-Insel Riems, Germany; ^4^ Faculty of Mathematics and Natural Sciences, University of Greifswald, Greifswald, Germany

**Keywords:** animal model, mycobacteria, tuberculosis, SARS-CoV-2, COVID-19, immunology, pathology, respiratory infection

## Abstract

Respiratory infections cause tremendous morbidity and mortality worldwide. Amongst these diseases, tuberculosis (TB), a bacterial illness caused by *Mycobacterium tuberculosis* which often affects the lung, and coronavirus disease 2019 (COVID-19) caused by the Severe Acute Respiratory Syndrome Coronavirus type 2 (SARS-CoV-2), stand out as major drivers of epidemics of global concern. Despite their unrelated etiology and distinct pathology, these infections affect the same vital organ and share immunopathogenesis traits and an imperative demand to model the diseases at their various progression stages and localizations. Due to the clinical spectrum and heterogeneity of both diseases experimental infections were pursued in a variety of animal models. We summarize mammalian models employed in TB and COVID-19 experimental investigations, highlighting the diversity of rodent models and species peculiarities for each infection. We discuss the utility of non-human primates for translational research and emphasize on the benefits of non-conventional experimental models such as livestock. We epitomize advances facilitated by animal models with regard to understanding disease pathophysiology and immune responses. Finally, we highlight research areas necessitating optimized models and advocate that research of pulmonary infectious diseases could benefit from cross-fertilization between studies of apparently unrelated diseases, such as TB and COVID-19.

## 1 Introduction

Animal models are essential for understanding disease pathophysiology in its complexity. Pinning down coordinated immune processes as well as the continuous host reaction to pathogen assault can only be achieved by investigating the infected host. Although controlled human infection models and human challenge trials have been advanced for flu (1), malaria (2), coronavirus disease 2019 (COVID-19) (3), and tuberculosis (TB) (4, 5), and studying infection in natural hosts in most circumstances is feasible for livestock, disease pathogenesis is studied in great detail in surrogate animals in experimental animal models. In such controlled settings pathogen entry, replication and transmission, immune responses, and pathology are elucidated unambiguously. Importantly, causality can be established in animal models and thereby such experimental approaches are instrumental for devising measures limiting pathogen transmission and for developing vaccines and therapies. The importance of animal models for vaccine testing should be emphasized. Here, animal models are without alternative (6) and should mimic the pathogenesis known in humans as closely as possible to increase transfer of the results to the human host (7). Standard laboratory animal models have been established to enable applications in many laboratories worldwide. Such models have been indispensable for the understanding, prevention and cure of two major respiratory infectious diseases: TB and COVID-19. We critically discuss experimental models in a comparative manner and highlight commonalities and differences in the context of these lung infections.

TB and COVID-19 are acquired respiratory infections which primarily affect the respiratory tract and are usually transmitted via aerosol droplets. TB represents one of the most ancient infectious diseases, a continuous threat to public health and currently among the top 10 causes of death worldwide (8). It was declared as a global emergency by the WHO in 1993 (9). TB is caused by genetically related microorganisms of the *Mycobacterium tuberculosis* complex (MTBC), with the human-adapted *M. tuberculosis* (Mtb) affecting mankind worldwide. COVID-19 represents the 21st century pandemic event and was declared as a global emergency by the WHO in 2020 (10). The global emergency phase was ended in May 2023, yet the WHO emphasizes that COVID-19 still remains a significant threat for human health^1^. It is caused by the severe acute respiratory syndrome coronavirus type 2 (SARS-CoV-2). Both infections are dynamic and provoke a spectrum of diseases and pathologies. Their causative agents, although taxonomically unrelated, undergo continuous adaptation to the human host. The potential to evade immunity has been observed promptly during the COVID-19 pandemic, for instance by the emergence of virus variants, whereas for TB resistance to available therapies is on the rise, as illustrated by heightened incidences of disease caused by drug-resistant mycobacteria. Mtb enters alveolar macrophages, rarely pneumocytes, and spreads to lung-resident and recruited macrophages, whereas SARS-CoV-2 primarily infects ciliated and alveolar epithelia (11). Although variable pathology is observed in TB and COVID-19, recent systems analysis of human cohorts revealed commonalities in immunopathogenesis (12). TB is characterized by unique lesions termed granulomas, whereas severe COVID-19 manifests as pneumonia. Given the preference for respiratory tissue, mammalian animal models have been developed for both infections. We discuss the experimental models employed for the study of each disease and emphasize advantages and limitations these models bring regarding disease pathophysiology and immune responses. Considering spectra of TB and COVID-19, we identify challenges related to improving or developing new animal models and propose purpose-oriented approaches which extend beyond conventional animal models. Finally, we elaborate on multi-species approaches and co-infections, as these are currently feasible and inspired by recent advances in high-resolution technologies.

## 2 Small animal models

Historically, small animal models, including rodents and leporids, were paramount for the identification of Mtb as the causative agent of TB and for the elucidation of TB pathogenesis (13). They continue to be implemented in preclinical TB research and have been equally instrumental for the accelerated progress achieved for COVID-19 vaccines. Although murine infection models are by far the most frequently used for TB and COVID-19 research, they reproduce some, but not all aspects of the human disease. Other rodent species, including rats, hamsters and guinea pigs, provide important insights into pathophysiological aspects of the two respiratory diseases that are not sufficiently covered by murine models. Each model organism offers particular advantages and bears certain limitations, which are detailed in the following sections.

### 2.1 Mouse models

Mice are easy to handle, accessible, inexpensive, and the broad availability of immunological and genetic tools makes them very attractive for preclinical investigations. Laboratory mice provide the most established and implemented animal model in SARS-CoV-2 as well as in TB studies (14, 15). Advantages and limitations of murine models in the two respiratory infections are presented in **Table 1**.

**Table 1:** Murine models for tuberculosis (TB) and coronavirus disease 2019 (COVID-19).

Mouse models	Tuberculosis		COVID-19	
	Advantage	Disadvantage	Advantage	Disadvantage
Inbred “resistant” (C57BL/6/Balb/c)	High reproducibility, availability of gene knock-out mice, long-term studies and kinetics, correlates of protection, immunological tools	Lack of human-like pathology (e.g. liquefaction, fibrosis), lack of relevant Mtb-induced cell types (e.g. multinucleated giant cells)	Availability of gene knock-out mice, long-term studies and kinetics, correlates of protection, immunological tools	Only mouse-adapted virus strains or specific SARS-CoV-2 variants (e.g. B.1.351), limited pathology
Inbred “susceptible” (C3HeB/FeJ, 129Sv, I/St, DBA/2)	Investigation of pathology, necrotic granuloma, correlates of susceptibility, drug testing	Limited number of knock-out mice available, no chronic or latent stage of disease	More severe pathology (129Sv) compared to C57BL/6, lung pathology (compared to K18-hACE2)	Only mouse-adapted virus strains or specific SARS-CoV-2 variants (e.g. B.1.351)
Outbred	Genetic diversity, microbiome diversity	Housing with inbred and pathogen-free mice difficult, reproducibility	N/A	N/A
Collaborative cross (CC) lines	Genetic diversity, gene association studies	Low reproducibility, expensive, resource intense	Genetic diversity, gene association studies	Require further genetic manipulation for usage as a model for COVID-19 (e.g. cross-breeding with K18-hACE2)
Humanized mouse	Reflection of human specific cell types or effector functions	Expensive, high variability, technology intensive, highly susceptible to attenuated strains (e.g. BCG)	Better reflect COVID-19 pathology, severe lung pathology, study of drug or antibody therapy	Expensive, high variability, technology intensive, cross-reactivity of human-mouse immune networks
Transgenic (K18-hACE2)	N/A	N/A	Robust, highly permissive model, suitable for all SARS-CoV-2 variants, excellent vaccine model	Limited lung pathology, brain pathology, some SARS-CoV-2 strains interact with human and mouse ACE2, expensive
Vector hACE2 delivery (AAV, Ad, LV)	N/A	N/A	Mild lung pathology (reflects COVID-19 in the majority of patients), suitable for vaccine studies, amenable for different mouse strains and genetic manipulations	Transient, low pathology (possible disadvantage for vaccine studies), immune response against vector
SARS-CoV-2 Mouse adaptation	N/A	N/A	Lung pathology (compared to K18-hACE2), can be used in combination with different mouse strains and genetic manipulations	Mouse adaptation might not reflect human isolates, new variants underrepresented

The various mouse models employed for the study of each disease are included, emphasizing on key advantages and limitations of each model. AAV, adeno-associated virus; Ad, adenovirus; COVID-19, coronavirus disease 2019; hACE2, human angiotensin-converting enzyme 2; K18, keratin 18 promoter; LV, lentivirus; N/A, not applicable; SARS-CoV-2, severe acute respiratory syndrome coronavirus type 2.

#### 2.1.1 Murine TB models

The experimental murine TB model has elucidated host fate upon natural infection which is achieved by aerosol exposure. It has unveiled the complexity of the kinetics of the infectious process in great detail. This model has also enabled mutual integration of host and pathogen traits in experimental studies. However, mice do not fully recapitulate TB pathology. Granuloma liquefaction, cavitation and fibrosis remain undetected in Mtb-infected mice, and hence murine TB is an imperfect disease model. This model allows to comprehensively study the immune responses to Mtb. Pulmonary anatomy and immune mechanisms in mice have a great degree of similarity to humans (16) which make them ideally suited for studying immune dynamics within tissues and for testing vaccine efficacy. Although mice are not natural hosts for Mtb and are generally tolerant to TB (17), they have proven instrumental for understanding some disease mechanisms.

Susceptibility of laboratory mice to Mtb depends on both host and bacterial features. Among the host factors, mouse genetics, age, sex, and immune status control TB outcome (18–21). The route of infection (22), inoculum size and bacterial genetics, e.g. Mtb lineages and virulence factors, impact as well on the course of TB. For instance, aerogenic exposure to the East/Asian Beijing strain HN878, in contrast to infection with the reference Euro-American strain H37Rv, triggers heightened susceptibility in C57BL/6 mice (23) and granulomatous lesions resembling human-like pathology (24). Various Mtb strains differ in propensity to infect myeloid cells (25), disseminate (26) or trigger inflammation (27). Mtb attenuation by deletion of the PhoP regulon (28), or deletion of entire virulence coding genomic regions, e.g. region of difference 1 (RD1) (29), or pathogenicity factors such as the early secreted antigenic target 6-kDa protein (ESAT-6) (30, 31) cause reduced pathology. Such studies have also unveiled a dominant role of nitric oxide in antimycobacterial immunity in the murine host, unlike in humans (32). In addition, they confirmed the relevance of subcellular pathogenicity events, such as cytosolic translocation of the bacilli, for Mtb pathogenicity during lung infection.

Research on TB immunology and pathology heavily relies on inbred and knock-out (KO) mouse strains and has recently been enriched by the addition of the collaborative cross (CC) lines (33, 34), diversity outbred (DO) (35–38) as well as humanized mice (39, 40). Whereas inbred animals and respective KO lines have permitted targeted characterization of host factors essential for the susceptibility to disease, genetic diversity has contributed to unbiased identification of host susceptibility or resistance traits. Of note, mice can be infected by various MTBC bacteria, and usage of transgenic knock-in models has facilitated analysis of particular cell types or molecules during infection (e.g. fluorescent-tagged reporters), or enabled targeted cell deletions (e.g. Cre-lox system). The transgenic mice used in TB research offer opportunities to decipher disease pathogenesis. They do not confer essential cell entry host factors to mycobacteria, a situation common for COVID-19 murine models where infection is usually abortive in wild type animals (see section 2.1.2).

TB outcome differs in various inbred mice which are classified as TB-resistant and TB-susceptible based on the time to death or bacterial outgrowth. TB-resistant mice, including C57BL/6 and Balb/c strains, control aerosol infection with relatively high doses (e.g. 500 colony forming units, CFU) of bacteria, do not develop typical granulomas and succumb rather due to aging. As such, they have been proposed as potential latency TB models (41). Studies in C57BL/6 mice have been critical for defining kinetics of the immune events post exposure, requirements for priming of adaptive immunity as well as kinetics and plasticity of T-cell responses in TB. Very early in infection alveolar macrophages support Mtb replication, as demonstrated in depletion studies (42), likely due to their metabolic imprinting towards oxidative phosphorylation (43) and anti-oxidant features (44). Elegant fate-mapping studies have highlighted that alveolar macrophages translocate into the lung interstitium (45) and Mtb gains access to less permissive glycolytic lung macrophages (43). Subcellular virulence factors, notably mycobacterial ESX1 secretion system (31), and host determinants of susceptibility, for instance phagosomal proteolysis (46) have been unveiled also *in vivo* in the context of macrophage plasticity in TB in C57BL/6 mice. Cell types conferring an Mtb-permissive environment, including lung monocyte-derived macrophages and dendritic cells (47), and neutrophils (48, 49), have been defined also in this murine model. Kinetics of T-cell responses (50) and the impact of their localization on disease outcome (51, 52) have been established in C57BL/6 mice receiving transgenic cells expressing an Mtb-specific T-cell receptor (TCR). T-cell depletion alone or combined with adoptive transfer of antigen-specific T-cells has indicated an essential role of CD4^+^ and CD8^+^ lymphocytes for TB control (53, 54) and highlighted Mtb escape strategies related to dominant epitopes and misplaced T-cells (55). Thus, C57BL/6 mice have substantially contributed to the delineation of immune events in primary TB. The major caveat of the C57BL/6 model lies in the lack of human-like pathology. Of note, application of ultra-low dose infection (ULD) (56) may render these mice amenable for pathology studies (57). Upon ULD, mice develop single, structured lesions upon inhalation of 1-3 Mtb CFU of the laboratory strain H37Rv. Organized granulomas have also been reported in C57BL/6 mice challenged with low-dose hypervirulent HN878 Mtb (24, 58). These murine models mirror, to some extent, human TB lesions, have organized granuloma-like lesions which contain foamy macrophages, develop central necrosis, yet still miss certain cellular components such as multinucleated giant cells and do not show fibrosis and calcification. A further utility of C57BL/6 mice has recently been reported. Intra-dermal Mtb infection resulted in localized spread of Mtb (59, 60), unlike systemic dissemination seen upon aerosol or intravenous challenge (22), and thus may represent a refined experimental model for latent TB infection (LTBI). Besides mechanistic understanding of immunity and pathology, C57BL/6 and Balb/c strains are also gold standards for chemotherapy studies and TB vaccine development.

TB-susceptible inbred mice encompass the C3HeB/FeJ, 129Sv (129S2/SvPas), I/St and DBA/2 mouse strains. C3HeB/FeJ mice are best suited for investigating pathology. Exposure of these mice to Mtb leads to the formation of well-formed, necrotic granulomas showing hypoxic regions (61), with liquefaction observable particularly upon i.v. challenge (62). Necrosis of Mtb-infected macrophages is controlled by the sst1 locus (63) and has been linked to the *intracellular pathogen resistance 1* (*Ipr1*) gene (64). A role for neutrophils in susceptibly to TB in C3HeB/FeJ by controlling lesion progression (36, 62), likely via type I interferon (IFN-I)-driven NETosis (65), has been reported. The similarities to lesion progression in humans (66–68) make this model useful for pathology and immunopathogenesis studies. DBA/2 mice show a fast TB course with bronchogenic dissemination (69). Their susceptibility is driven by neutrophils (70) and limited accumulation of regulatory T-cells (Treg) within infected tissue (71). This phenotype is shared by the TB-susceptible inbred strain I/St (72, 73), which unlike their A/Sn counterparts cannot control TB. 129Sv (129S2) mice succumb early during TB (74) with extensive lung damage. Their susceptibility to TB is uncoupled from *natural resistance-associated macrophage protein 1* (*Nramp1*) allele gene polymorphism (75). Mtb-triggered lethality is due to early neutrophil recruitment (74), heightened necrotic cell death (76), and likely Mtb-driven and acetyl-coenzyme A dependent foamy cell differentiation (77). TB-susceptible mice are suitable for deciphering host traits which favor a poor outcome in TB. They are also helpful for testing drugs and host-directed interventions given the development of lesions, notably well-structured granulomas containing transformed cell types, and environments, such as hypoxia and necrosis, characteristic of active TB.

Understanding of the immune control of TB has been nurtured by failed immunity in KO and immunodeficient mice which mirror catastrophic human genetic defects. Examples are mice with full or cell-type specific deletion of IFN-γ (78) or TNF-α (79, 80). Just as reported in humans with mendelian susceptibility to mycobacterial disease (81) or on suppressive anti-TNF-α immunotherapy (82) these cannot control Mtb infection or reactivate LTBI, respectively. Since immunity can be investigated within organs, KO mice also enriched knowledge about *in situ* roles of host factors. For instance, IFN-γ was shown to regulate neutrophil apoptosis (83) and TNF-α to regulate lesion stability by signaling in myeloid and lymphoid cells (80). Mice lacking lymphocytes (Rag2 KO and Rag2/γc KO) have demonstrated essential yet differential roles of T- and NK-cell derived IFN-γ in TB control (84). KO mice have supported reverse translation investigations in TB, as exemplified for miRNAs. For instance, miR-223 is enriched in human TB lesions and susceptibility of miR-223 KO has been linked to the regulation of IL-6, as well as of CCL3 and CXCL2, during acute disease (85). Thus, various KO mice have supported the understanding of TB pathogenesis at a molecular level. In contrast, susceptibility of certain KO lines has not translated to observations in human TB. Some examples are heightened mortality associated with mice lacking the adaptors MyD88 (86) and CARD9 (87). Failed models with observations distinct from human data are also exemplified by mice lacking NADPH oxidase subunits (88, 89) or indolamin-2,3-dioxygenase (90). These mice do not show a strong phenotype in TB despite susceptibility being linked to deficiency in these pathways in humans (68, 91). The inbred features of the most common murine models may contribute to such discrepancies. In this context, CC lines and DO mice may better mirror genetic diversity of the human host. CC lines have uncovered genetic loci associated with uncontrolled infection, including IFN-γ-independent phenotypes (33). DO models have confirmed that neutrophils are detrimental in progressive TB and highlighted roles of neutrophil chemoattractants in this process (36). Since mice and humans show variabilities in immune components, for instance cell abundance (humans belong to neutrophil-high species) or molecular constituents (mice lack granulysin and CD1-type-1 molecules), humanized murine models have been generated (39, 40). Their use is restricted due to financial and technological constraints as well as variability in immune reconstitution and persistence of a mixed human-mouse tissue environment. However, their usage could be critical for addressing co-infection of Mtb with viruses requiring human host factors for entry such as human immunodeficiency virus (HIV). Without doubt, KO models have substantially enriched the knowledge about immune cells and immune pathways in TB and provided causality proofs for disease pathogenesis. Embracing genetic diversity by usage of CC line and DO mice offers unique opportunities for mechanistic studies and may provide new ways to guide TB prophylaxis (92).

Collectively, murine models for TB are diverse and offer a spectrum of options to choose from (see **Table 1**). Experimental tools and feasibility of gene editing in mice, which permit cell fate mapping and tracing, will continue to support immunological research. There are yet several limitations related to the usage of mice in TB. Besides the drawback regarding TB pathology, mice are not suitable for transmission studies. They have been extensively used as models for primary TB. However, unlike humans, mice promptly allow Mtb dissemination to distal sites following aerogenic infection. Efforts to develop murine models for post-primary TB have been undertaken (41) and require additional evaluation.

#### 2.1.2 Murine COVID-19 models

Inbred laboratory mouse strains such as Balb/C and C57BL/6 are not susceptible to ancestral (B.1) SARS-CoV-2 infection. With the emergence of SARS-CoV-2 variants (e.g. Alpha (B.1.1.7), Beta (B.1.351), and Gamma (P.1)) with extensive mutations in the spike protein, particularly the N501Y mutation, laboratory mouse strains became susceptible to infection and virus replication, although without showing significant pathology (93). However, the inbred mouse strain 129S2 develops clinical disease and has been employed to assess the efficacy of monoclonal antibodies and vaccines (94, 95). Two major approaches have been pursued to amend the murine model for COVID-19 study: genetic engineering of mice for expression of the human ACE2 (hACE2) receptor protein, and adaptation of SARS-CoV-2 to enter murine cells via endogenously expressed receptors (96, 97). A comprehensive summary of the frequently used genetically manipulated murine models as well as adapted virus strategies that substantially contributed to reproduce key characteristics of SARS-CoV-2 infection has been provided recently (98). For comparative evaluation we integrate the murine COVID-19 models with TB models and highlight benefits and disadvantages for each model and infection (**Table 1**).

The most commonly used K18-hACE2 model, where hACE2 is expressed under control of the human keratin 18 promotor, in addition to the murine ACE2, appears to be the most susceptible COVID-19 model reported to date using human SARS-CoV-2 isolates (99). This model has contributed to the clarification of disease pathophysiology. For instance, it provided evidence for SARS-CoV-2 invasion of sustentacular cells as the cause of subsequent anosmia (100). As inflammation drives severity of COVID-19 in humans, details on dynamics of inflammatory responses obtained in this mouse model could be valuable for the design of therapies. The K18-hACE2 mouse resembles severe COVID-19 disease (101), developing cytokine storm (102), prompt accumulation of immune cells within infected lung (103), loss of plasmacytoid dendritic cells (104) and alveolar macrophages paralleled by accumulation of monocyte-derived macrophages (105). The contribution of host genetics to inflammation control has been further evaluated using collaborative cross (CC) x K18-hACE2 F1 progeny mice (106). In this model survival was associated with early IFN-I expression and production of proinflammatory factors. Similarly, disease severity was driven by CXCR6 and CCR9 in a comparable mouse model approach (107). The K18-hACE2 model has been also useful to demonstrate the relevance of lymphoid cell depletion, which together with the impaired antigen presenting cells/T-cell axis, is a specific feature of severe SARS-CoV-2 infection (108). Furthermore, evidence for the protective roles of T-cells was demonstrated by the fact that vaccination with immunodominant T-cell epitopes provided partial or even full protection in K18-hACE2 mice in the absence of neutralizing antibodies (109, 110). Comparative RNAseq analysis (human vs. mouse) has revealed that at the broad level of immune responses and inflammation pathways, highly overlapping patterns between the two species exist suggesting that the K18-hACE2 mouse model emerges as a representative and relevant animal model of COVID-19 (111). It remains yet unclear whether innate immunity alone could under particular circumstances, for instance low inoculum, eliminate the virus in these transgenic mice. A disadvantage of this model is that it does not mirror mild disease, and interference of signaling from both murine and human ACE2 adds an additional layer of complexity when investigating SARS-COV-2 variants which bind the murine receptor (104). Further, the K18-hACE2 mouse model also has the disadvantage of hACE2 expression in the brain of the transgenic animals. The severity of the disease and the reason for humane endpoints are therefore usually the artificial occurrence of a severe infection of the brain with encephalitis (101, 112). Brain invasion has been demonstrated in humans (113), it is though not a common manifestation of COVID-19 (114). Of note, aerosol delivery in contrast to intranasal challenge bypasses brain involvement (115), suggesting that the route of infection may be relevant for the phenotype of the K18-hACE2 murine model. The K18-hACE mouse model has been essential for vaccine research and its preclinical value is impressive. The critical role of the murine models is highlighted by the fact that mRNA vaccine preparations were extensively tested in the mouse model before licensing in the U.S. under Emergency Use Authorization. Additionally, next generation SARS-CoV-2 vaccines covering multivalency or mucosal application have been similarly evaluated in mouse models [i.a (116–119)].

Adenovirus-, lentivirus- or adeno-associated virus-driven transient hACE2 expression in the murine lung has also been established multiple times in different laboratories [e.g (97, 120, 121)]. However, the virus-induced expression comes with the disadvantage of potential induction of unspecific inflammatory responses, non-uniform expression of hACE2 in the lung epithelium and interference with vector-based vaccines (121). Nevertheless, it has been utilized to study COVID-19 pathology and for preclinical vaccine investigations, including mechanism of action studies. The pathology in this model is restricted to the respiratory tract, with milder disease and in most cases self-resolving inflammation (121, 122). Using this model, it has been shown that IFN-I responses are associated with inflammation and myeloid cell infiltration, but not with SARS-CoV-2 control (122, 123). The mild and localized pathology, along with the possibility to induce hACE2 expression in KO strains, have enabled to study mechanisms of SARS-CoV-2 clearance in naïve and vaccinated animals with different genetic backgrounds. These studies have confirmed the essential role of the adaptive immunity for resolution of inflammation and viral clearance (124). Furthermore, protection of neutralizing antibodies has been confirmed in this animal model (120).

Another approach that allows the use of standard laboratory mice and, more importantly, genetically modified mice, is to adapt SARS-CoV-2 to the mouse (96). These viral strains are therefore particularly suitable for studies in specific KO mouse lines. Thus, the age and sex dependency of human disease severity could be shown with an adapted ancestral SARS-CoV-2 strain (125, 126). However, such adaptations must be carried out separately for different virus variants which do not naturally infect mice and thus are disadvantageous due to the extensive time required for adaptation.

All mouse models come with a substantial drawback related to viral transmission. Even humanized and genetically modified mice are unable to transmit the virus to contact animals (127). Of note, recent investigations in a neonatal K18-hACE2 mouse model have reported virus transmission in a SARS-CoV-2 variant specific manner (128) and such promising observations require validation.

**Figure 1:**
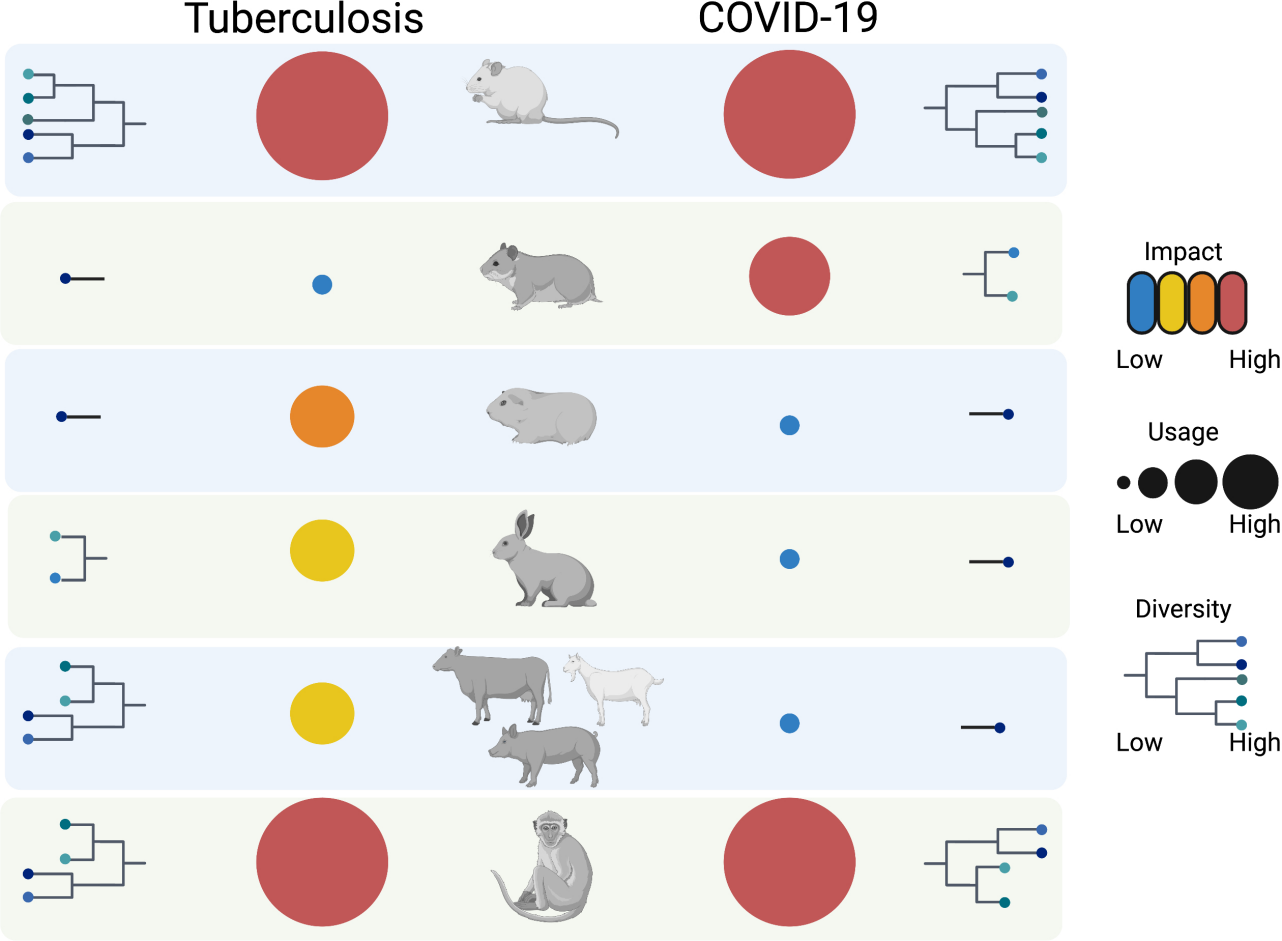
Overview of animal models for TB and COVID-19. The animal models used for the two respiratory infections are diversified and range from mouse to non-human primates. Whereas murine models show the highest diversity, guinea pigs, rabbits, hamsters and livestock show applicability for one of the two diseases. Non-human primates, just like mice, have the biggest impact in terms of knowledge gain with the former ones having the greatest translational value. All animal models have unique benefits and cumulatively contribute to the study of TB and COVID-19, and have potential to cross-fertilize understanding of other respiratory diseases. The figure was generated using the illustration software BioRender (BioRender.com).

The murine models used for TB and COVID-19 differ substantially, primarily due to the distinct natural susceptibility of mice to Mtb and SARS-CoV-2 (**Table 1**). For both infections, mouse models are not amenable for investigating transmission and generally have limitations due to dissemination of infection at distal sites as well as at recapitulating human pulmonary pathology. Nonetheless, they are suitable for the mechanistical understanding of immune responses and thus have been extensively employed for vaccine studies. The diversification of the mouse models in TB during the last decade is remarkable (**Figure 1**), and attempts to employ systems approaches for vaccine discovery (129) further emphasize their value in pre-clinical research. Whereas transgenic knock-in mice have been essential for the progress of COVID-19 vaccines, such strains have rather targeted utility in TB. Irrespective of the peculiarities of the murine models, in both infections experimentation in mice has permitted evaluation of biological processes at subtissular and molecular scale and have advanced interventions.

### 2.2 Rat models for COVID-19 and TB

The rat is the animal species of choice in the pharmaceutical industry for pharmacokinetic and toxicological studies. Wistar rats are also generally employed in immunization studies given their broad availability, easy handling, defined physiology and potential to obtain larger samples compared to mice. They are infectible by selected SARS-CoV-2 variants, such as B.1.1.7 (130), but have not been used as a model for COVID-19. Instead, Wistar rats have been essential for investigating the pharmacokinetics of the lipid-nanoparticles used to formulate COVID-19 mRNA vaccines [EPAR – Comirnaty] (129–132). A limitation for vaccine studies in this model is the insufficient knowledge about SARS-CoV-2-induced pathology and the lack of appropriate immunological tools to monitor immune responses (e.g. T-cell responses) after vaccination and challenge.

Rats are generally susceptible to Mtb (132), and they have been used to distinguish bacteriostatic or bactericidal properties of investigational compounds (133). In the rat model, the decrease of T-cell reactivity to ESAT-6 has been proposed as a correlate of therapeutic efficacy (134) which principally sheds light on the maintenance of high-level T effector cell populations. Various rat models, including American cotton rats, Lewis rats, Wistar rats, and Sprague-Dawley rats, develop granulomatous lesions which do not liquefy (132, 135, 136), and thus human TB pathology is not fully mirrored.

Rats have proven valuable for TB diagnostic purposes, particularly in poor resource settings: African giant pouch rats have been trained to detect Mtb in sputum samples (137). Mycobacterial volatile organic compounds are detected by rats which recognize Mtb across different genotypes and discriminate it from related bacteria, including *M. avium* subsp. hominissuis or *M. intracellulare* (137). Although in this case the animal model does not immediately contribute to the understanding of TB pathophysiology, the approach has drawn attention to an entirely unexplored universe of small volatile bacterial compounds, and has brought forth a diagnostic method to detect TB in high burden regions with limited access to molecular diagnostics. Thus, the rat model could be useful to develop electronic nose devices for TB detection.

### 2.3 The guinea pig model for TB

Guinea pigs are resistant to SARS-CoV-2 (138), but are highly susceptible to TB. In his pioneering experiments Robert Koch used guinea pigs and rabbits to prove that a pure Mtb culture causes the disease (13). In the 1950s their susceptibility to TB prompted scientists to use guinea pigs as living air samplers to demonstrate aerial dissemination of mycobacteria (139). They not only take up mycobacteria by inhalation, but also expectorate them like humans and thus are amenable to transmission studies. Recently, guinea pigs have been used to reveal sulfolipid-1 as the activating factor for nociceptive neurons to trigger cough (140). The course of infection in guinea pigs varies with the Mtb strain and the initial dose, but invariably animals succumb to Mtb infection. After logarithmic growth in the lungs, Mtb loads remain stable over many weeks. Ultimately, the bacteria re-enter a logarithmic growth phase and this regularly coincides with the humane end point (141). Besides aerosol exposure also parenteral routes of infection are used. For example, the intramuscular route and the degree of generalized systemic dissemination has been used to assess the virulence of different Mtb isolates (142, 143). For batch potency testing of bovine tuberculin it is laid down in the corresponding monography of the European Pharmacopoeia that guinea pigs shall be sensitized by deep intramuscular injection of live *M. bovis* before defined amounts of the control and the test batch of the tuberculin are intradermally injected (Eur Ph 01/2008:0536). In guinea pigs, initial Mtb replication is confined to the site of entry, yet bacteria disseminate via lymphatic flow presumably by dendritic cells reaching the draining lymph node (144). Secondary to lymphadenitis, which is often a manifestation of TB in children and consistently developed in guinea pigs (144), there is systemic generalization and hematogenous spread to other organs. Ultimately, hematogenous reseeding of the lungs may occur which leads to progressive infection and tissue destruction. At these different tissue levels granulomatous infiltrates occur that develop to large, caseous, necrotizing granulomas in unsensitized animals (144). However, it is important to note that granulomas in guinea pigs barely show liquefaction and cavitation (145, 146). Such lesions are the hallmark of post-primary TB and, upon infection, can prominently be observed in pre-sensitized rabbits (see 2.4). In guinea pigs, granulomas rather reproduce primary lesions in humans. Accordingly, guinea pigs are not a suitable model to study mycobacterial latency (145). The vast, necrotizing lesions develop in the absence of preformed T-cell immunity and are probably due to early recruitment and decay of granulocytes (147). In the presence of antigen-specific T-cells, guinea pigs show fewer granulomas that are better structured and contain significantly smaller necrotic areas. This correlates with reduced bacterial burden (141). Hence, guinea pigs have been widely used to stringently test new vaccine candidates against TB (148–153). The observed protective effect can be achieved by immunizing guinea pigs with protein antigens, but also with mycobacterial lipids (154). In this context, it is of note that guinea pigs express a functional CD1-type1-system. This is another hallmark that distinguishes them from murine rodents and resembles humans (155–157). CD1 molecules are characterized by a deep, hydrophobic antigen binding groove which enables accommodation and presentation of long-chained lipids to T-cells. In contrast to CD1d-restricted NKT cells, the lymphocytes that recognize their antigen in the context of CD1-type-1-molecules bear a variable αβ-T TCR and truly belong to the adaptive immune system. They can be primed and develop an immunological memory (158). Because mycobacteria express a rich repertoire of glycolipids, lipoglycans and lipopeptides, which all represent or harbor potential CD1-ligands (159), the CD1-T-cell axis has always been of interest to TB vaccinologists. Due to their susceptibility to Mtb and the natural expression of CD1, guinea pigs are particularly well-suited to study the contribution of lipid-reactive T-cells to defense against Mtb (160). Accordingly, efforts have been undertaken to study the protective role of lipid-reactive T-cells (154, 160), but additional animal studies are required to better understand the complex interaction between mycobacterial lipids and the host’s adaptive immunity (161). Guinea pigs are also suitable for evaluation of diagnostic skin tests and thus are an essential animal model for assessing delayed-type hypersensitivity to mycobacterial cognates (141, 162). They are in addition an indispensable model for testing antimycobacterial compounds (163–165).

A drawback of the guinea pig model remains the scarcity of immunological tools and the lack of genetically modified strains. However, a number of guinea pig-specific, monoclonal antibodies have become available in recent years (166, 167). In addition, molecular screening techniques including gene arrays, qRT-PCR and classical immunological stimulation assays, have been developed to study guinea pig immune responses in more detail (168–170).

### 2.4 The rabbit model for TB

Rabbits can be infected with high doses of the ancestrally derived SARS-CoV-2, but are not suitable as model animals for COVID-19 because of their low susceptibility (171). On the other hand, they allow studying clinical features of TB since rabbits are relatively resistant to Mtb compared to *M. bovis* (172). Infection with Mtb strains can lead to a latent course of disease that can be reactivated by immunosuppressive drugs (173). Using this model, it has been shown that rapid innate immunity involving in particular an early activation of NK cells is essential for an early control of exponential bacterial growth. It has also been shown that T-cell activation is dampened once bacterial growth is controlled, leading to spontaneous latency (174). By contrast, infection with *M. bovis* results in extended lung tissue destruction ultimately leading to cavity formation (175). The rabbit model is amenable to closely reproduce post-primary TB. Animals develop cavities similar to lesions in humans, in a process that involves congestion of bronchioles, massive multiplication of mycobacteria and extensive, allergic necrotizing tissue destruction and depends on Mtb strains, previous sensitization, and host genotype (176). Sensitization of rabbits by multiple injections of heat-killed *M. bovis* in incomplete Freund’s adjuvant and subsequently instillation of viable Mtb by bronchoscopy directly into the lung triggers cavitation (177). This approach has led to a better understanding of the role of matrix-metallo-proteases in TB cavity formation. Ability to induce cavitation depends also on Mtb strains, with hypervirulent W-Beijing Mtb causing cavities, while less virulent strains including CDC1551 rather trigger LTBI (173, 178). The outcome of Mtb exposure can be studied in rabbits and has unveiled that early innate inflammatory responses, inoculum size and bacillary aggregation facilitate progressive TB and development of pathology rather than establishment of LTBI (178, 179). Zonation of pro- and anti-inflammatory regions within granulomas, primarily due to variable abundancies of distinct eicosanoid species, are similar in rabbits and humans (180). TB pathology in rabbits, and specifically occurrence of cavities, reproduce this stage of the disease that is most critical for successful antibiotic treatment (181). Currently, the rabbit model has become instrumental to study the biodistribution of new and of well-known antimycobacterial compounds, such as rifampicin (182) and pyrazinamide (183). As for guinea pigs, lack of immunological reagents limits vaccinology studies in rabbits. The model has also limitations with regard to the clinical manifestation of TB. Moreover, genetic editing of rabbits is in its infancy and the high costs compared to rodent models restrict usage of the rabbit model to specific scientific questions.

### 2.5 Hamster models for COVID-19

Hamsters, including the golden Syrian hamster (*Mesocricetus auratus*), are susceptible to TB, but have not been extensively used for the study of this disease (184). They are naturally highly susceptible to SARS-CoV-2 infection (185–187). Experimental intranasal inoculation with SARS-CoV-2 results in a transient, self-limiting, epitheliotropic infection of the lungs with almost complete elimination of the virus within two weeks. Certain dwarf hamsters (e.g. the Roborovski dwarf hamster) are even more susceptible and usually die or have to be euthanized after SARS-CoV-2 challenge (187). In the Syrian hamster, SARS-CoV-2 infection is restricted to sites containing both ACE2 receptor protein and TMPRSS2 protease (188). Interestingly, the infectious dose 50 for Syrian hamsters is defined to be only five infectious particles, making the hamster a sensitive model for SARS-CoV-2 infectivity assessment (189). In this model, host factors have been investigated and variable influence of age on disease severity has been reported (189, 190). Syrian hamsters are also suitable to explore sex differences in the pathogenesis of SARS-CoV-2 and vaccine-induced immunity and protection (191, 192). Transmission to direct contact hamsters as well as airborne-based transmission occurs in this animal model [i.a (193, 194)]. Furthermore, the concept of super-spreading has been modelled in the Syrian hamster model (194, 195). These findings strengthen the superior value of the hamster model over other SARS-CoV-2 models for virology and disease pathogenesis studies. Besides utility in deciphering acute host responses to SARS-CoV-2, the Syrian hamster offers an alternative for modeling of long COVID-19. Despite the lack of detectable infectious virus hamsters exhibit altered long term systemic responses (196).

Although immunological tools are limited, SARS-CoV-2–specific T- and B-cells have been evaluated in a longitudinal study in infected and recovered hamsters (197). Adoptive T-cell transfer reduces virus loads and facilitates rapid induction of SARS-CoV-2–specific B-cells, demonstrating that both lymphocyte populations mutually contribute to protection in hamsters. Studies applying single-cell RNA and protein profiling have substantiated the utility of the hamster model for deciphering immune events in moderate COVID-19. Similar to human COVID-19 patients, early proinflammatory responses from lung-residing monocyte-derived macrophages have been detected in SARS-CoV-2 infected hamsters (198). The animals develop inflammatory profiles akin to the cytokine storm observed in humans (196). *In situ* accumulation of cytotoxic T-cells and release of IgM antibodies occur prior to viral elimination (198). Golden Syrian hamsters reproduce also the vasculopathy observed in human patients, including involvement of neutrophil extracellular traps (NETs) (199) which is observed in severe human cases (200). The hamster has been, and continues to be, instrumental for both COVID-19 vaccinology and therapy. Its translation value seems to exceed that of mice (15). More recently the hamster has provided mechanistic insights into the Th-2 basis of vaccine-associated enhanced respiratory disease (201) and emphasized the value of tissue-resident memory T-cells (202) in protection against SARS-CoV-2 conferred by distinct live-vaccines.

Overall, the hamster is one of the most significant animal models for the study of SARS-CoV-2 pathogenesis and for vaccine development. In addition to usage of modern single-cell technologies, immunological tools are increasingly being developed for this species with the prospect of advancing hamster studies in the future.

## 3 Large animal models

Livestock species and non-human primates (NHP) are natural hosts for MTBC, with the latter ones being also prone to SARS-CoV-2 infection. Similarities to humans with respect to the anatomy of the respiratory tract and the structure of the lung, for instance lung lobulation, as well as commonalities in organization and functionality of the immune system are notable. The evolutionary relationship with humans confers large animal models additional assets and unique model values.

### 3.1 Non-human primate models

NHP have been essential for elucidating SARS-CoV-2 and TB disease pathogenesis as well as for vaccine studies. Three different NHP, Rhesus Macaques (RM), Cynomolgus Macaques (CM) and African Green Monkeys (AGM) have been primarily used for both pathogens with the rational that they are genetically and immunologically closely related to humans.

#### 3.1.1 Non-human primates in TB

In the 1960s and 1970s RM were used for the first time in TB research for vaccine and drug efficacy testing. For two decades RM and CM have offered substantial novel insights into pathology, immunology, vaccine and therapies for TB. Nowadays the NHP model is considered the most relevant for translational human TB research (203).

Depending on the dose (10^1^-10^5^ CFU), Mtb strain (e.g. Erdman, H37Rv, CDC1551), and route of infection (intravenous, intratracheal or aerosol) RM and CM reflect the full TB spectrum (acute, LTBI and re-activation of LTBI) including all stages of human-like granuloma (204). NHP and human mature, adaptive granulomas, are structured into necrotic cores surrounded by layers of macrophages and lymphocyte zones (205), including immunocompromised microenvironments (68). Akin human lesions (206), NHP granulomas contain tertiary lymphoid structures with key roles in anti-mycobacterial immunity (207). Progression to active TB can be monitored in NHP by longitudinal MRI or PET-CT scans which correlate with bacterial burden and inflammation (208). Such clinical measurements revealed that even under non-clinical disease (e.g. LTBI) NHP lungs contain active, necrotic lesions and sterilized healing lesions at the same time (209), an observation which has been confirmed with similar methods in humans (210). RM are more susceptible to Mtb infection than CM, with RM showing increased pathology and progression to disease compared to CM (211). Evidence for variable baseline of anti- and pro-inflammatory status of the myeloid compartment resulting in increased anti-inflammatory responses in RM after Mtb infection compared to CM pro-inflammatory responses has been provided (212) and likely additional factors underlying diverging susceptibility exist.

Since the whole spectrum of human TB can be observed in NHP models, correlates of protection or susceptibility have been singled out by comparing progressor versus non-progressor animals and by comparing individual progressing versus sterile granulomas from the same animal (213). Treatment of NHP with an antibody against TNF-α leads to increased disease progression in line with observations in humans (214, 215). Similarly, co-infection of NHP with Mtb and Simian immunodeficiency virus (SIV) leads to active disease with pathological features comparable to HIV-1 co-infection in humans (216, 217). Consistent with human TB and many mammalian models, CD4+ T-cells play an essential role in protecting NHP against development of active TB (218, 219). Single-cell transcriptomic signatures of different granulomas from the same individual lung sample revealed that healing or sterile granuloma were associated with IFN-γ/IL17 producing Th1 CD4+ T-cells (213). However, T-cells alone do not seem to be sufficient to control Mtb infection in NHP and humans. Tertiary lymphoid structures (e.g. inducible Bronchus-Associated Lymphoid Tissues (iBALT) or granuloma-associated lymphoid tissue (GrALT)) are significantly associated with non-progressors for active TB (220). A recent study of these GrALT structures has revealed that Mtb-specific B-cells induce T follicular helper cells (Tfh cells) to promote such protection, while depletion of B-cells or impairment of Tfh cells would lead to reduction of GrALT and bacterial growth (207). These findings now require further investigations in human TB and highlight the power of the NHP model to advance knowledge about human TB.

Protection of NHP models against disease progression provided by BCG depends on the route of vaccination, the NHP model, the Mtb challenge strain and dose. Overall, intradermal BCG vaccination of NHP provides variable protection against pulmonary TB which might reflect BCG efficacy in humans (221–224). BCG appears to be more efficient when delivered via aerosol in low dose (225, 226) or when administered intravenously (224, 227). NHP have also been used extensively to test safety and efficacy of preclinical and clinical TB vaccine candidates (228). The vaccine candidate M72/AS01E, which showed 54% efficacy in a human clinical phase 2 trial (229) also showed efficacy in the CM model (230).

Thus, the NHP model greatly contributes to the understanding of TB pathology, correlates of protection and vaccine efficacy. NHP recapitulate active, latent TB, and TB reactivation, and are amenable to longitudinal studies with serial sampling, including imaging, as well as study of TB comorbidities. Limitations of this model are the high housing costs, ethical concerns and shortage of RM and CM for experimental studies. In addition, the variability in route of infection, inoculum, Mtb strain and NHP model lead to heterogenous outcomes, making it challenging to select the most appropriate experimental setup for translational studies.

#### 3.1.2 Non-human primates in COVID-19

The ACE2 receptor for SARS-CoV-2 in NHP is identical to hACE2 (231), which is an advantage over other mammalian models. Pathogenesis, vaccine and therapeutic studies have been primarily performed in RM (232), CM (233) and AGM (234) almost simultaneously and immediately after the start of the pandemic. In general, experimental infection resembles mild and/or moderate COVID-19 in humans. It reflects a mild to moderate disease course (235) including lung pathology, viral replication in the upper respiratory tract, vascular involvement including thrombosis (232) and more severe clinical symptoms in aged NHP (236). A direct comparison of RM and CM after SARS-CoV-2 challenge has demonstrated that both models are comparable in the clinical course of infection, viral replication, as well as humoral and cellular immune response (237).

The moderate clinical course in the NHP model allows investigations regarding the correlates of protection. The acute phase and viral replication peak at around 2-4 days post infection and virus genomic RNA and clinical signs decline rapidly afterwards (238). The dynamics of the viral burden are mirrored by influx of neutrophils, dendritic cells and monocyte/macrophage populations into the lung which peak around day 3 and resolve one week later (239). The inflammatory response in the lung of NHP seems dominated by infiltrated monocyte-derived macrophages and is required for clearance of infected pneumocytes and inflammation afterwards (240). This indicates that in NHP the innate immune system likely contributes to the control of virus replication and resolution of inflammation. In line with this, the decline of virus replication and inflammation was associated with IFN-I activated myeloid cells before the induction of adaptive immunity (241). The established immunity protects RM against re-infection, which is similar to observations in humans (242). However, in this case it is most likely mediated by humoral and cellular responses in the upper respiratory tract (243, 244).

NHP have been extensively used as a preclinical model for all currently licensed vaccines against COVID-19 (245). In this context NHP proved to be relevant to investigate correlates of protection and mechanisms of action of COVID-19 vaccines. Systemic neutralizing antibody titers have been found to provide protection induced by the mRNA-1273 vaccine in non-human primates and humans (246, 247). In case of declining antibody titers over time SARS-CoV-2-specific CD8+ T-cell responses provide additional protective immunity and T-cell responses correlate with protection (level of SARS-CoV-2 sgRNA) in RM vaccinated with mRNA-1273 (246). In summary, NHP serve as an excellent model for moderate human COVID-19 cases as well as for investigations of correlates of protection and vaccine efficacy. However, cost restraints and ethical concerns along with a shortage of RM for experimental studies (248) require complementation by other models for studying COVID-19 pathology and for vaccine development.

### 3.2 Livestock models for TB

Large livestock species have been tested for their susceptibility for COVID-19. However, SARS-CoV-2 does not establish productive infection, nor does it disseminate in farm species such as cattle, goats and pigs (249). In contrast, livestock species are natural hosts and are therefore used as models for human TB. While Mtb is a human-adapted strain, other members of this family such as *M. orygis*, *M. caprae* and *M. bovis* are zoonotic pathogens. The main reservoir for these MTBC members are livestock species, including cattle, goats and pigs (250–252). However, these bacteria can infect humans and cause undistinguishable pathology compared to Mtb-driven disease, yet more often extra-pulmonary disease (253, 254). Of note, Mtb can infect livestock, for instance cattle, but usually does not induce a comparable pathology. Especially under experimental conditions cattle, goats and pigs can eradicate Mtb (255–257). Therefore, livestock species may serve as a model for human TB to investigate pathology (e.g. *M. bovis*) and correlates of protection (Mtb).

Natural MTBC infections in cattle, goats and pigs cause granulomas of all stages as described in humans, including necrotic lesions containing extracellular mycobacteria (256, 258, 259), and well-contained fibrotic encapsulated granulomas (260). *M. bovis*-induced granulomas in cattle are characterized by a strong expansion of IFN-γ-producing CD4+ T-cells and *M. bovis*-specific B lymphocytes (261, 262). Like in humans, *M. bovis*-induced activation of CD8+ T cells seems low compared to CD4+ T cells, but their presence might support Th1 response (263). The lesions developed in minipigs encompass caseous, fibrotic to calcified granulomas within the lungs and lymph nodes. Granulomas progress to encapsulation in pigs. This fibrous cuff develops in close proximity to the fibrotic capsule which anatomically limits the lung lobules and seems to contribute to the containment of infection (260). Thus, the lobular partitioning of the lung which is seen in livestock and in NHP, but not in rodents, may significantly restrict bacillary dissemination. Of note, in pigs, bacilli can be transmitted from infected to naïve animals, possibly due to development of cavities (264). Tissue features and pathogen transmissibility underscore the value of pigs for transmission studies (265).

In all species, macrophages and their precursors (e.g. monocytes) are the main intracellular niche for *M. bovis* or Mtb (266). Bovine monocytes show functional and developmental similarities to monocyte subsets in humans (267). In line with monocyte analogies in man and cattle, bovine monocyte-derived macrophages are a niche for intracellular growth of *M. bovis*, respond with a pro-inflammatory response and contribute to early granuloma formation (268, 269). Likewise, neutrophils have been found in humans, mice, and cattle to be recruited early during infection with MTBC bacteria (270). Some anti-mycobacterial defense mechanisms might be species-specific with bovine myeloid cells being equipped with a high number of antimicrobial peptides, variable granules and pattern recognition receptors (PRRs) (271).

Experimental infection of cattle with *M. bovis* leads to an early development of pulmonary lesions and development of necrotic granulomas rich of bacteria, neutrophils and giant cells already 30 days post challenge (272). However, progression to clinical disease might take several years (273). Whether *M. bovis* becomes latent during this time and can be reactivated like in humans is not well understood (274). Strikingly, experimental infection of pigs, goats and cattle with Mtb results in recovery of low bacterial numbers and Mtb-associated lesions from infected animals (255–257). These findings indicate that Mtb is attenuated in other species. In pigs, using a high dose i.v. challenge model, induction of systemic IFN-y responses was similar in *M. bovis* versus Mtb infected pigs suggesting that the abundance of Th1 responses does not correlate with disease outcome (257, 275). Strong Th1 responses also have been observed in miniature pigs aerogenically challenged with Mtb (260, 265). Systemic delivery of *M. bovis* results in early onset of clinical disease in piglets and development of TB granulomas in the wall of the meningeal vessels (275). Occurrence of brain pathology makes piglets appealing for modeling childhood meningeal TB, a disease form which is difficult to model in other experimental animals. The bovine immune system may tolerate low abundant Mtb or develop distinct T-cell responses against Mtb to restrict its replication. For example, T-cell responses against the Mtb/*M. bovis* antigen Rv3879c have been only detected in *M. bovis*-infected, but not in Mtb-infected cattle. This supports the hypothesis that the T-cell repertoire could differ and therefore also recognition and/or activation of infected macrophages by CD4+ T-cells (255, 276). Host tropism and lack of adaptation to ruminants likely confer to Mtb a limited replication advantage, and presumably immune-competent cattle and other mammalian species are dead-end hosts eliminating the human-adapted Mtb. Resistance of cattle to Mtb may also rely on differences in very early responses of lung cells to Mtb versus *M. bovis*. Variability in activation of the cytosolic DNA-sensing pathways (277) and subsequent IFN-I responses (278), as well as regulation of cytokines or receptors for pathogens (279) have been reported. In addition, Mtb and *M. bovis* seem to reside in different compartments in bovine and human macrophages and only *M. bovis* and *M. bovis*-derived MPB70 trigger multinucleation of macrophages (269). However, roles of the multinucleated giant cells in the resistance phenotype and in other species, such as pigs and goats, remain to be demonstrated. The two MTBC members could trigger distinct responses in other myeloid cells, too, or may differently alter immune responses, cell networking or tissue remodeling. In-depth characterization of protective immune responses in cattle, goats, pigs as models for human TB could unmask novel correlates of protection in natural hosts and inform rational design of therapeutics in humans.

Vaccine efficacy testing in several studies with BCG in cattle was similarly inconclusive to efficacy studies in humans. Like in humans, BCG supports the induction of an IFN-y and CD4+ T-cell response, however it does not seem to prevent granuloma formation and disease progression in cows compared to calves (280), bearing similarities to age-imprinted protection in humans. Recent studies in calves have unveiled that BCG delivery via aerosol trained circulating monocytes, yet left antimycobacterial responses of alveolar macrophages unchanged (281). BCG-driven *ex vivo* training of cattle monocyte is similar to human counterparts, however aerogenic immunization seems inefficient at remodeling mucosal immune cells. In pigs, a study from 1932 suggested that BCG vaccination induces small healing lesions, but only limited protection against infection with Mtb (282). Recent data from this model have highlighted its value specifically for understanding neonatal and juvenile responses to BCG. Piglets receiving BCG show development of effector CD4+ lymphocytes and maintain frequencies of CD8+ T-cells constant over time. However, higher abundancies of activated monocytes persist after Mtb challenge (264). Whether the monocyte changes are associated with trained innate immunity, as known in human neonates, and have a critical role in protection remains to be investigated. Likewise, there is limited experimental data using BCG vaccinated goats. However, one report suggested that BCG has only a limited protective efficacy after challenge with *M. caprae* (259). More recent advanced goat models using video endoscopy for infection via intrabronchial spray inoculation (283) have demonstrated the relevance and suitability of goats for vaccine studies using BCG and new clinical candidates (284, 285). Considering that BCG is the only licensed vaccine against TB it still remains the gold standard when testing new vaccine concepts. BCG vaccination in cattle, pigs and goats might reflect outcome in humans, and therefore these are useful models for novel preclinical vaccine concepts. However, further studies in large livestock species are required.

Ruminants and pigs bring benefits for TB studies by offering unique opportunities to investigate disease susceptibility and resistance in natural hosts. Whereas experimentation in cattle is difficult due to their size and the high expenses related to the maintenance of infected animals for longer periods of time in high containment laboratories, goats offer a viable alternative given their smaller size, lower costs and easier maintenance. The immunology toolbox for ruminants is still limited. Immunological reagents available for pigs exceed those for ruminants. Moreover, pigs are smaller, largely available and relatively easy to sample and handle. Availability of outbred and inbred lines, as well as recent advances in gene editing make them appealing for TB research. Apparent limitations due to inversion of lymph nodes or immunological peculiarities related to lymphocyte subsets are compensated by similarities with regard to the organization of the immune system in pigs and humans (286) and the extensive experience from other medical fields, such as transplantation. Furthermore, pigs could be exploited for neonatal immunology in the context of BCG immunization and offer an experimental model for meningeal TB.

## 4 Perspectives

Animal models offer opportunities to investigate host responses in great detail and under controlled conditions, considering the interlinked reactivity of various organs over time. Describing currently used animal models for TB and COVID-19 it becomes obvious that there is no ideal model (**Figure 1**). Each model comes with benefits and limitations and only their purpose-oriented utilization or usage of multiple models can adequately clarify a specific scientific question and advance interventions. Since both infections affect the respiratory tissue, cross-fertilization from established animal models for TB and COVID-19 appear natural. Certainly, advances in investigational methodologies, for instance for analysis of immunity in Mtb-infected NHP, have been swiftly translated from TB to COVID-19 (241). For other models, such as mice, translation of models from TB to COVID-19 was limited due to abortive viral infection in standard laboratory strains. Nonetheless, we envisage that these models may contribute to the elucidation of counter-regulation in TB and COVID-19 as it happens in co-infection. Mtb may change the host landscape for SARS-CoV-2 and vice-versa, and such cross-regulations are critical for the co-infected human host. Regarding interventions, the extensive expertise of BCG in pre-clinical research has paved the way for understanding whether its heterologous effects contribute to protection against SARS-CoV-2. Importantly, knowledge gain from coinfection studies or the value of BCG-triggered trained immunity for an emerging viral disease, notably COVID-19, may be valid for other pneumonias and could serve for rapid action in case of a future pandemic episode.

Animal models have been employed to decipher effects of SARS-CoV-2/Mtb coinfection, which is critical because both pathogens persist in the human and wildlife populations. Natural infections with each pathogen currently have been reported in certain species, although coinfection has been evaluated solely for humans (**Table 2**). Concerns about the severity of COVID-19 in the LTBI population or the risk of TB reactivation subsequent to infection with SARS-CoV-2 were raised shortly after COVID-19 emergence, and co-infection has been associated with higher mortality rates (299–301). Studies analyzing human cohorts report that subclinical and active TB may increase the risk of severe COVID-19 due to circulating myeloid subpopulations found in severe COVID-19 or impaired antiviral activity (12, 302, 303). Regarding effects of the viral pathogen on the control of bacterial replication, SARS-CoV-2 leads to reduced frequencies of Mtb-specific CD4+ T-cells which may facilitate TB progression (304). Of note, dysregulation of IFN-I is observed in both infections (305–307). The relevance of such cellular subsets and phenotypes as well as of the immune pathways relevant for TB outcome has been demonstrated in animal models (74, 308, 309). In line with clinical presumptions, the murine hepatitis virus, which is a mouse-adapted coronavirus, reactivates Mtb in a dormant mouse model using a streptomycin-auxotrophic mutant bacterial strain (310). Co-infection studies in mice addressing effects of TB on SARS-CoV-2 infection outcome so far have led to inconclusive results. K18-hACE2 mice chronically infected with Mtb limit SARS-CoV-2 loads (311) or become resistant to SARS-CoV-2 infection, presumably due to the strong Th1 milieu (312). These disparities may be due to imperfect modeling of the co-infection in the mouse and also to the spectra of disease for each infection. Thus, experimental co-infection of natural hosts of both pathogens might be more suitable for such investigations (**Table 2**). Since Mtb and SARS-CoV-2 infect multiple species aside from their host of choice, attempts to model them in other animal models or multiple species could be helpful. Following this approach, epidemiological observations from the human population could be explored to define molecular determinants controlling inflammation and cell death pathways which may co-regulate host-responses to both pathogens (313). A priority should be the elaboration of solutions for bottlenecks in mirroring diseases at various stages and certainly this becomes complex in co-infection and co-morbidity scenarios which are often associated with TB and COVID-19.

**Table 2:** Currently known hosts with the potential of coinfection.

Species	MTBC strain	SARS-CoV-2 strain	References
Humans and non-human primates	*M. bovis* and Mtb	Ancestral and all variants	Hlavsa et al., 2008 (287) Wu et al., 2020 (288) Lerche et al., 2008 (289) Qiu et al., 2023 (290)
White-tailed deer	*M. bovis*	Alpha, Delta, Omicron	Vandergrift et al., 2022 (291) Marques et al., 2022 (292)
Minks, ferrets	*M. bovis*	Ancestral	Virtanen et al., 2022 (293) Shi et al., 2020 (294) Gupta et al., 2022 (295) Oude Munnink et al., 2021 (296)
Felidae	*M. bovis*	Ancestral	Giraldo-Ramirez et al., 2021 (297) Miller et al., 2019 (298)

The animal species and families from which virulent mycobacteria belonging to the *Mycobacterium tuberculosis* complex (MTBC) as well as SARS-CoV-2 have been isolated are included. Details on the MTBC and virus strain and references reporting detection of the pathogen in respective animal species are provided.

Modeling of potential unspecific benefits of BCG in surrogate animals generally has produced consistent results. Whereas systemic BCG protects mice from influenza A virus lethality (314), it does not protect hamsters from SARS-CoV-2 and its effects were inconsistent in K18-hACE2 transgenic mice (314, 315). The disparities in mice may stem from the usage of various BCG strains and variable study protocols. Aerosol delivery of BCG leaves the course of SARS-CoV-2 infection unchanged in RM (316). These results are overall supportive of observations from a large clinical trial: BCG (Denmark strain) did not reduce the risk of COVID-19 (317). Thus, the power of employing multiple animal models for devising interventions has been further substantiated in the context of BCG immunization for heterologous protection and represents a lesson learned from the COVID-19 pandemic.

For both TB and COVID-19 there are still knowledge gaps which should be addressed using experimentation in animal models. Current models do not fully allow to define determinants of TB latency, triggers of disseminated disease, mechanisms underlying tolerance to disease and molecular regulators of TB reactivation. Similarly, understanding factors which drive the development of long COVID-19, as well as multisystemic inflammatory syndrome in children (MIS-C), is a priority. In the context of disease resolution, both for TB and COVID-19 reparatory processes as well as regulators of tissue sequelae remain largely elusive. Furthermore, the cellular and molecular basis of TB vaccine efficacy in young individuals, particularly neonates and infants are still not understood. Addressing these topics requires fit-for-purpose models and likely cross-species analysis. The multi-host disease feature and lung localization in both infections, along with the recent progress in single-cell technologies offer opportunities. The scientific community has initiated parallel deep profiling in multiple experimental models, and guidance for respiratory infections has recently been provided (318). Such agnostic approaches can be harnessed for the development of therapies and vaccines. Fit-for-purpose examples of animal models are juvenile pigs for early life conditions such as MIS-C and meningeal and miliary TB. Studies in juvenile pigs could also model vaccination in human neonates. Pigs already have provided robust results for disease pathogenesis, unveiling subtissular localization of virus-specific CD8^+^ resident memory T-cells (319) and interventions, for instance mode of action of monoclonal antibodies (320) or various vaccine platforms (321), for flu. Pigs could also be a model for acute coronavirus infection (322). For the study of chronic COVID-19, engraftment of mice with human hematopoietic and stem cells (323) offers an alternative as these animals show lung pathology and fibrosis observed in severely ill patients.

Development of new animal models could clarify questions which cannot be addressed using available models. Acknowledging translatability issues from mice to humans, novel “wildlings” mice which combine the natural microbiome with genetic tractability of C57BL/6 mice emphasize the validity of this approach for reproducibility and translatability of immunological findings in biomedical studies (324). This model has not been applied yet in infectious disease research, but given the universality of housing mice it could be readily implemented. Studies of pathogen transmission are key for TB and COVID-19, however reliable and accessible animal models are scarce. The ferret is particularly suitable due to the anatomy of larger intranasal structures (325). SARS-CoV-2 infection foci with oligofocal pattern have been detected using a 3D microscopy approach in ferret conchae (326). Moreover, in characterizing SARS-CoV-2 variants of concern, it provides an additional model to investigate *in situ* viral competition (327) showing that, for instance Omicron BA.1 was no longer able to replicate in the presence of evolving variants (328). Recent studies have reported that ferrets successfully transmit Mtb and develop TB pathology (295), thus extending the model value of ferrets also to a bacterial respiratory infection. Housing and handling ferrets in high-containment laboratories requires adequate training and space, making experimentation feasible only at selected institutions. Other examples of novel model animals, particularly amenable to decipher disease tolerance, are bats. Bats harbor multiple viruses without showing signs of disease, and experimental challenge with SARS-CoV-2 has resulted in productive infection in the absence of disease (325). Understanding the basis of the resilience in bats could advance therapies, and high-end technologies have been applied recently to unmask the immune landscape in bats at steady state and during infection (329, 330). Access to bat colonies is restricted to only few research facilities worldwide and the value of bats does not rely in phenotyping a disease stage, but rather in recapitulating resilience in disease-free individuals. Thus, novel animal models with peculiar features are available for respiratory infections and the examples presented herein are not exhaustive. They could all contribute to uncovering the pathophysiology of maladaptive immune responses, including hyperinflammation and immunosuppression, as well as of the extensive lung destruction and dysfunction detected in TB and COVID-19.

In conclusion, for the understanding of infectious diseases as well as for testing of vaccines or therapeutics, targeted and well-considered use of animal models is still indispensable. It must be pointed out that it is essential to follow the 3R concepts to reduce, replace and refine usage of animals in experimental research. These ethical-driven approaches represent the foundation of animal experimentation around the world, and it is conceivable that in some cases newer systems, such as three-dimensional cell culture or organoids, will continue to proof themselves to be able to replace some of the animal testing. When considering two unrelated pathogens, as in our example with Mtb and SARS-CoV-2, it is noticeable that similar questions arise, which are then analyzed with the appropriate model in each case. Therefore, an important step is the selection of animal models to be used according to the available infrastructure, tools and scientific needs. However, common issues such as paucity of immunological reagents in non-murine models require solutions. Here, joint interdisciplinary (bacteriology and virology) and intersectoral (human and veterinary medicine) efforts are necessary to increment the value of non-conventional animal models and address societal needs, and state-of-the-art single cell technologies offer opportunities.

## Author contributions

Conceived and designed the paper: BC and AD. Drafted the paper: BC, MBa, DH, MBe, AD. Revised the paper for critically intellectual content and review final manuscript: BC, MBa, DH, MBe, AD.

## Footnotes

1 WHO. WHO Director-General's opening remarks at the media briefing, Vol. 2023. (2023).
